# Gutta Percha as a Base for Artificial Teeth

**Published:** 1852-01

**Authors:** 


*Gutta Percha as a Base for Artificial Teeth.*—We have received a communication from Robert Parsons, surgeon dentist, of York, England, informing us that a Mr. Turner had obtained a patent for "fixing artificial teeth upon a preparation of gutta percha." He also states that he has a set in his possession, the entire base of which is composed of the same material, which had been worn for some time, and made by his brother Mr. J. H. Parsons, dentist, of Halifax, who had made others during his residence at Liverpool. He does not, however, regard this material as suitable for the purpose, and says, "it can never supercede well adapted gold plates, or carefully sea horse ivory foundations."

Some six or eight months previously to the receipt of the communication here referred to, we received a letter from a professional gentleman residing in New England, who informed us that he had succeeded in making a preparation of gutta percha which he had used as a base for artificial teeth. He also informed us that in the cases in which he had used it, it had fully realized his most sanguine expectations, promising that after he should have tested it sufficiently, and if it should prove to be really valuable for this purpose, to make the discovery known to the profession. He moreover sent us a small piece of the preparation, and, we believe, he is still engaged in trying to improve it. We hope he will soon give us an opportunity of laying the results of his experiments before the profession.

